# Poly[[tetra-μ_3_-acetato-hexa-μ_2_-acetato­diaqua-μ_2_-oxalato-tetra­lanthanum(III)] dihydrate]

**DOI:** 10.1107/S1600536811038037

**Published:** 2011-09-30

**Authors:** Wen-Jing Di, Shao-Min Lan, Qun Zhang, Yun-Xiao Liang

**Affiliations:** a State Key Lab. Base of Novel Functional Materials and Preparation Science, Faculty of Materials Science and Chemical Engineering, Ningbo University, Ningbo, Zhejiang, 315211, People’s Republic of China

## Abstract

The title compound, {[La_4_(CH_3_CO_2_)_10_(C_2_O_4_)(H_2_O)_2_]·2H_2_O}_*n*_, exhibits a two-dimensional layered structure with the oxalate and acetate ligands acting as bridges. The asymmetric unit contains two crystallographically independent lanthanum(III) ions, half of an oxalate ligand, five acetate ligands, one coordinated water mol­ecule and one uncoordinated water mol­ecule. The coordination numbers of the two La ions are 9 and 10. Adjacent layers of the structure, which extend parallel to (100), are linked by O–H⋯O hydrogen bonds and are also held together by van der Waals inter­actions between the CH_3_ groups of the acetate anions.

## Related literature

For properties of lanthanide compounds with metal-organic framework structures, see: Zhu *et al.* (2006 ▶); Deng *et al.* (2009 ▶); Bünzli & Piguet (2005 ▶); Zhang *et al.* (2008 ▶). For metal oxalates, see: Kustaryono *et al.* (2010 ▶); Roméro & Trombe (1999 ▶); Yu *et al.* (2006 ▶); Ohba *et al.* (1993 ▶). For lanthanide oxalates obtained from oxalate-containing starting materials, see: Zhang *et al.* (2009 ▶); Trombe *et al.* (2005 ▶). For lanthanide oxalates with oxalate formed in the course of the synthesis by decomposition of organic compounds or other unconventional reactions, see: Koner & Goldberg (2009 ▶); Li *et al.* (2003 ▶); Min & Lee (2002 ▶); Mohapatra *et al.* (2009 ▶). For oxidation of acetate to oxalate, see: Zieliński (1983 ▶). For La—O bond lengths, see: Trombe & Roméro (2000 ▶); Deng *et al.* (2009 ▶). For coordination modes of acetate groups, see: Zhang *et al.* (2009 ▶); Dan *et al.* (2006 ▶); Koner & Goldberg (2009 ▶); Mazurek *et al.* (1985 ▶). 
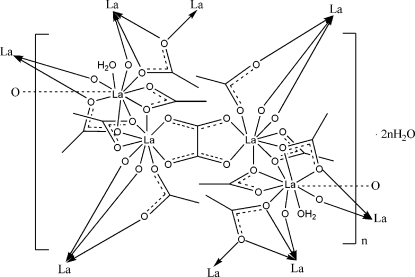


## Experimental

### 

#### Crystal data

[La_4_(C_2_H_3_O_2_)_10_(C_2_O_4_)(H_2_O)_2_]·2H_2_O
*M* *_r_* = 653.08
Monoclinic, 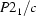

*a* = 9.4139 (19) Å
*b* = 13.310 (3) Å
*c* = 16.087 (3) Å
β = 103.10 (3)°
*V* = 1963.2 (7) Å^3^
*Z* = 4
Mo *K*α radiation
μ = 4.36 mm^−1^
*T* = 295 K
0.19 × 0.18 × 0.18 mm

#### Data collection

Rigaku R-AXIS RAPID diffractometer
Absorption correction: multi-scan (*ABSCOR*; Higashi, 1995 ▶) *T*_min_ = 0.454, *T*_max_ = 0.456
14917 measured reflections
3432 independent reflections
3185 reflections with *I* > 2σ(*I*)
*R*_int_ = 0.023

#### Refinement

*R*[*F* ^2^ > 2σ(*F* ^2^)] = 0.019
*wR*(*F* ^2^) = 0.042
*S* = 1.12
3432 reflections
245 parameters
H-atom parameters constrained
Δρ_max_ = 0.56 e Å^−3^
Δρ_min_ = −0.40 e Å^−3^

### 

Data collection: *RAPID-AUTO* (Rigaku, 1998 ▶); cell refinement: *RAPID-AUTO*; data reduction: *CrystalStructure* (Rigaku/MSC, 2002 ▶); program(s) used to solve structure: *SHELXS97* (Sheldrick, 2008 ▶); program(s) used to refine structure: *SHELXL97* (Sheldrick, 2008 ▶); molecular graphics: *ORTEPII* (Johnson, 1976 ▶) and *DIAMOND* (Brandenburg & Putz, 2008 ▶); software used to prepare material for publication: *publCIF* (Westrip, 2010 ▶).

## Supplementary Material

Crystal structure: contains datablock(s) I, global. DOI: 10.1107/S1600536811038037/qk2021sup1.cif

Structure factors: contains datablock(s) I. DOI: 10.1107/S1600536811038037/qk2021Isup2.hkl

Additional supplementary materials:  crystallographic information; 3D view; checkCIF report

## Figures and Tables

**Table 1:** Hydrogen-bond geometry (Å, °)

*D*—H⋯*A*	*D*—H	H⋯*A*	*D*⋯*A*	*D*—H⋯*A*
O13—H13*A*⋯O14	0.82	1.84	2.654 (4)	176
O13—H13*B*⋯O9^i^	0.82	2.01	2.831 (3)	176
O14—H14*B*⋯O9^ii^	0.87	2.12	2.921 (4)	152.9
O14—H14*A*⋯O5^iii^	0.86	1.90	2.761 (4)	174.3

Symmetry codes: (i) 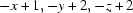; (ii) 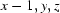; (iii) 
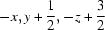
.

**Fig. 1.**
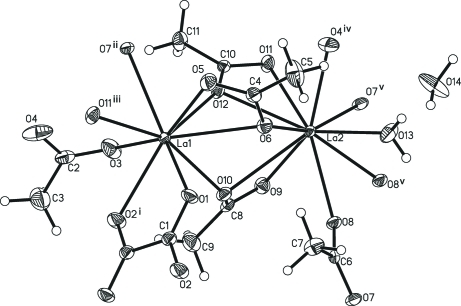
The asymmetric unit of (I), showing the atom-labelling scheme. Displacement ellipsoids are drawn at the 30% probability level and H atoms are shown as small spheres. [Symmetry codes: (i) –x+1, –y+1, –z+2; (ii) x, –y+3/2, z–1/2; (iii) –x+1, y–1/2, –z+3/2; (iv) –x+1, y + 1/2, –z+3/2; (v) –x+1, –y+2, –z+2.

**Fig. 2.**
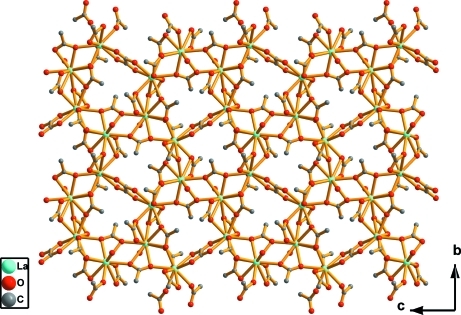
A view, along the a axis, showing the two-dimensional layered structure. H atoms and noncoordinated water molecules between the layers have been omitted for clarity.

**Fig. 3.**
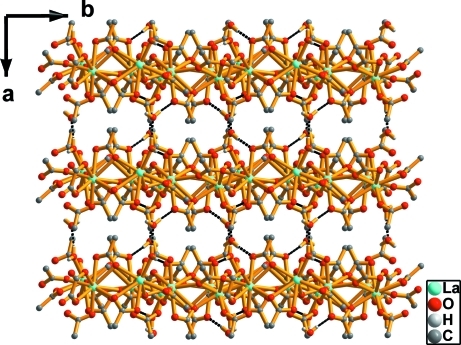
The three-dimensional structure of (I), viewed down the c axis. Hydrogen bonds are indicated by dashed lines.

## supplementary crystallographic information

### Comment

Recently, lanthanide metal-organic frameworks have attracted considerable attention due to their interesting properties, such as porosity (Zhu *et al.*, 2006), luminescence (Deng *et al.*, 2009; Bünzli & Piguet, 2005) and magnetism (Zhang *et al.*, 2008). The interest in mixed-ligand lanthanide oxalates is due to: first, lanthanide ions have large size, high and variable coordination numbers and flexible coordination environments; second, oxalate, as a bisbidentate ligand, has a strong coordination ability to metal ions (Kustaryono *et al.*, 2010; Roméro & Trombe, 1999; Yu *et al.*, 2006; Ohba *et al.*, 1993). Most of the known lanthanide oxalates were prepared by hydrothermal reactions of various oxalate salts or of mixtures containing free oxalic acid and other reagents (Zhang *et al.*, 2009; Trombe *et al.*, 2005), while in a few instances the oxalate was generated by chance via an *in situ* decomposition of organic reagents (Koner & Goldberg, 2009; Li *et al.*, 2003; Mohapatra *et al.*, 2009). In one case a metal-assisted reduction of CO_2_ was invoked to explain an unexpected oxalate formation (Min & Lee, 2002).

We report here the synthesis and crystal structure of a new lanthanum oxalate acetate, where the oxalate was formed *in situ* under hydrothermal conditions presumably by an redox reaction of copper(II) acetate under concomitant formation of Cu_2_O (see Experimental). When the reaction was carried out without copper(II) acetate, different products were obtained. Oxidation of acetate to oxalate has been reported (Zieliński, 1983).

As shown in Fig. 1, the asymmetric unit of (I) contains two La ions, a half oxalate anion, five acetate groups, one coordinated water molecule, and one noncoordinated water molecule. The two crystallographically independent lanthanum atoms, La1 and La2, are nine- and ten-coordinated by oxygen atoms. The La1 ion is coordinated by seven acetate oxygen atoms (one acetate in chelating fashion) and two oxygen atoms O1 and O2 of a chelating centrosymmetric oxalate group. The La—O bond distances vary from 2.476 (3) to 2.727 (2) Å (see supplementary materials). The La1—O bond distances of the oxalate group are 2.505 (2) Å and 2.519 (2) Å. The La2 ion is coordinated by nine acetate oxygen atoms (three acetate groups in chelating fashion) and by a water molecule (H_2_O13) in terminal position. The La2—O bond distances vary from 2.427 (3) to 2.802 (2) Å. The La—O bond distance of the coordinating water molecule is 2.517 (3) Å. All La—O bond distances of (I) are in the normal range for La(III) ions (Trombe & Roméro, 2000; Deng *et al.*, 2009). In the crystal structure of (I), each centrosymmetric bisbidentate oxalate ligand bridges two neighbouring La1 ions. The acetate groups have three different coordination modes: Firstly, the acetate group is µ_3_-η_2_-η_2_-bridging (the group chelates one La and links with each oxygen to a second and third La). Such coordination has been observed previously (Zhang *et al.*, 2009; Dan *et al.*, 2006). Secondly, the acetate group chelates one La ion and binds with one of its two O to a second La. Such coordination mode of lanthanide acetates has been previously observed (Koner & Goldberg, 2009; Dan *et al.*, 2006). Thirdly, an acetate ion bridges two metal ions with each oxygen bonded to one La. Such coordination mode has also been reported previously (Mazurek *et al.*, 1985).

As shown in Fig. 2, the title compound possesses a two-dimensional polymeric layered structure parallel to (100). The layer contains as a characteristic feature 10-membered oval rings of La atoms linked by the acetate groups. Each of these rings is subdivided in two centrosymmetric halves by a central oxalate bridge, which reinforces the layer. While the central parts of the layers are formed by the La ions and the carboxyl groups of acetate and oxalate anions, the outer parts of the layers are formed by the CH_3_ groups of the acetate groups and by the La-coordinating water molecules. Therefore, perpendicular to (100) the layers are mutually held together by van der Waals contacts between the CH_3_ groups and by the noncoordinating water molecule H_2_O14, each of which links two layers via one accepted and two donated O—H···O hydrogen bonds (Table 1). This leads to the formation of a three-dimensional supramolecular network shown in Fig. 3.

### Experimental

Copper acetate monohydrate (>98%, Shanghai Zhenxing Reagent Factory), lanthanum acetate hydrate (99.99%, Crystal Pure Reagent Co., Ltd. Shanghai), borax(>99.5%, Chemical Co., Ltd. Wuxi Jiani), acetic acid (>99.5%, Sinopharm Chemical Reagent Co., Ltd.) were used without further purification.

The mixture of copper acetate monohydrate (0.097 g, 0.5 mmol), lanthanum acetate hydrate (0.318 g, 1 mmol), borax (0.381 g, 1 mmol), acetic acid (0.2 ml) and deionized water (7 ml), was placed in a 23 ml Teflon reactor and stirred for 20 min in air, then heated at 453 K for 5 d, followed by cooling to room temperature at a rate of 5K/h. Colorless transparent X-ray quality single crystals of compound (I) and red single crystals of Cu_2_O were obtained, which were used for X-ray diffraction analysis.

### Refinement

The H atoms of the methyl groups were located from the difference Fourier map and were constrained to ride on their parent atoms with C—H = 0.96 Å, and with *U*_iso_ = 1.5 *U*_eq_(parent atom). The water H atoms were placed in geometric positions and refined with a riding model, O—H = 0.82 Å for the coordinated water, O—H ≈ 0.86 Å for the noncoordinated water.

### Crystal data

**Table:**

[La_4_(C_2_H_3_O_2_)_10_(C_2_O_4_)(H_2_O)_2_]·2H_2_O	*F*(000) = 1244
*M**_r_* = 653.08	*D*_x_ = 2.210 Mg m^−^^3^
Monoclinic, *P*2_1_/*c*	Mo *K*α radiation, λ = 0.71073 Å
Hall symbol: -P 2ybc	Cell parameters from 3432 reflections
*a* = 9.4139 (19) Å	θ = 3.0–25.0°
*b* = 13.310 (3) Å	µ = 4.36 mm^−^^1^
*c* = 16.087 (3) Å	*T* = 295 K
β = 103.10 (3)°	Block, colourless
*V* = 1963.2 (7) Å^3^	0.19 × 0.18 × 0.18 mm
*Z* = 4	

### Data collection

**Table:**

Rigaku R-AXIS RAPID diffractometer	3432 independent reflections
Radiation source: fine-focus sealed tube	3185 reflections with *I* > 2σ(*I*)
graphite	*R*_int_ = 0.023
ω scans	θ_max_ = 25.0°, θ_min_ = 3.0°
Absorption correction: multi-scan (*ABSCOR*; Higashi,1995)	*h* = −11→11
*T*_min_ = 0.454, *T*_max_ = 0.456	*k* = −15→15
14917 measured reflections	*l* = −19→18

### Refinement

**Table:**

Refinement on *F*^2^	Secondary atom site location: difference Fourier map
Least-squares matrix: full	Hydrogen site location: inferred from neighbouring sites
*R*[*F*^2^ > 2σ(*F*^2^)] = 0.019	H-atom parameters constrained
*wR*(*F*^2^) = 0.042	*w* = 1/[σ^2^(*F*_o_^2^) + (0.014*P*)^2^ + 3.2282*P*] where *P* = (*F*_o_^2^ + 2*F*_c_^2^)/3
*S* = 1.12	(Δ/σ)_max_ = 0.002
3432 reflections	Δρ_max_ = 0.56 e Å^−^^3^
245 parameters	Δρ_min_ = −0.40 e Å^−^^3^
0 restraints	Extinction correction: *SHELXL*, Fc^*^=kFc[1+0.001xFc^2^λ^3^/sin(2θ)]^-1/4^
Primary atom site location: structure-invariant direct methods	Extinction coefficient: 0.00041 (8)

### Special details

**Table:**

Geometry. All esds (except the esd in the dihedral angle between two l.s. planes) are estimated using the full covariance matrix. The cell esds are taken into account individually in the estimation of esds in distances, angles and torsion angles; correlations between esds in cell parameters are only used when they are defined by crystal symmetry. An approximate (isotropic) treatment of cell esds is used for estimating esds involving l.s. planes.
Refinement. Refinement of *F*^2^ against ALL reflections. The weighted *R*-factor *wR* and goodness of fit *S* are based on *F*^2^, conventional *R*-factors *R* are based on *F*, with *F* set to zero for negative *F*^2^. The threshold expression of *F*^2^ > σ(*F*^2^) is used only for calculating *R*-factors(gt) *etc*. and is not relevant to the choice of reflections for refinement. *R*-factors based on *F*^2^ are statistically about twice as large as those based on *F*, and *R*- factors based on ALL data will be even larger.

### Fractional atomic coordinates and isotropic or equivalent isotropic displacement parameters (Å^2^)

**Table:**

	*x*	*y*	*z*	*U*_iso_*/*U*_eq_	
La1	0.49400 (2)	0.626091 (13)	0.826328 (11)	0.01839 (7)	
La2	0.43860 (2)	0.927787 (13)	0.868342 (11)	0.01851 (7)	
O1	0.3617 (3)	0.57173 (19)	0.93738 (15)	0.0334 (6)	
O2	0.3667 (3)	0.4876 (2)	1.05838 (16)	0.0388 (6)	
O3	0.7514 (3)	0.6156 (2)	0.81474 (18)	0.0488 (8)	
O4	0.7413 (4)	0.4615 (3)	0.76263 (18)	0.0636 (10)	
O5	0.2212 (3)	0.64689 (19)	0.75919 (16)	0.0370 (6)	
O6	0.2988 (3)	0.76927 (17)	0.84850 (14)	0.0282 (5)	
O7	0.4657 (3)	0.89359 (17)	1.16394 (13)	0.0291 (6)	
O8	0.4577 (3)	0.91183 (17)	1.02914 (13)	0.0267 (5)	
O9	0.7158 (3)	0.90241 (17)	0.93121 (15)	0.0299 (6)	
O10	0.6023 (3)	0.75914 (17)	0.93639 (13)	0.0272 (5)	
O11	0.5756 (3)	0.95427 (17)	0.73455 (15)	0.0335 (6)	
O12	0.5291 (3)	0.80015 (16)	0.76449 (14)	0.0275 (5)	
O13	0.2161 (3)	1.0002 (2)	0.90822 (16)	0.0474 (8)	
H13B	0.2400	1.0278	0.9549	0.071*	
H13A	0.1270	0.9998	0.8919	0.071*	
O14	−0.0721 (3)	1.0087 (3)	0.8562 (2)	0.0745 (12)	
H14B	−0.1085	0.9687	0.8892	0.050*	
H14A	−0.1241	1.0501	0.8208	0.050*	
C1	0.4214 (4)	0.5171 (2)	0.9990 (2)	0.0258 (7)	
C2	0.8037 (4)	0.5293 (3)	0.8105 (2)	0.0347 (9)	
C3	0.9496 (5)	0.5054 (4)	0.8655 (3)	0.0589 (13)	
H3A	0.9749	0.4372	0.8556	0.088*	
H3B	0.9467	0.5134	0.9244	0.088*	
H3C	1.0212	0.5500	0.8520	0.088*	
C4	0.1955 (4)	0.7242 (3)	0.7973 (2)	0.0268 (8)	
C5	0.0446 (5)	0.7640 (3)	0.7823 (3)	0.0584 (13)	
H5A	0.0469	0.8360	0.7797	0.088*	
H5B	0.0011	0.7436	0.8281	0.088*	
H5C	−0.0119	0.7381	0.7293	0.088*	
C6	0.4177 (4)	0.8653 (2)	1.08777 (19)	0.0206 (7)	
C7	0.3107 (5)	0.7813 (3)	1.0678 (2)	0.0392 (9)	
H7A	0.2863	0.7697	1.0074	0.059*	
H7B	0.2241	0.7985	1.0867	0.059*	
H7C	0.3528	0.7215	1.0966	0.059*	
C8	0.7178 (4)	0.8098 (3)	0.95129 (19)	0.0257 (8)	
C9	0.8593 (5)	0.7623 (3)	0.9952 (3)	0.0455 (10)	
H9A	0.8595	0.6930	0.9788	0.068*	
H9B	0.8705	0.7669	1.0558	0.068*	
H9C	0.9385	0.7967	0.9789	0.068*	
C10	0.5884 (4)	0.8614 (2)	0.72301 (19)	0.0229 (7)	
C11	0.6738 (4)	0.8243 (3)	0.6616 (2)	0.0365 (9)	
H11A	0.6250	0.8430	0.6047	0.055*	
H11B	0.6818	0.7525	0.6655	0.055*	
H11C	0.7695	0.8536	0.6752	0.055*	

### Atomic displacement parameters (Å^2^)

**Table:**

	*U*^11^	*U*^22^	*U*^33^	*U*^12^	*U*^13^	*U*^23^
La1	0.02665 (12)	0.01320 (10)	0.01550 (10)	0.00021 (7)	0.00519 (7)	0.00054 (6)
La2	0.02568 (12)	0.01433 (10)	0.01519 (10)	−0.00034 (7)	0.00396 (7)	−0.00131 (6)
O1	0.0374 (16)	0.0368 (15)	0.0278 (13)	0.0104 (12)	0.0111 (11)	0.0146 (11)
O2	0.0411 (17)	0.0468 (17)	0.0327 (14)	0.0106 (13)	0.0172 (12)	0.0183 (12)
O3	0.0396 (18)	0.060 (2)	0.0522 (18)	0.0180 (15)	0.0222 (14)	0.0200 (14)
O4	0.067 (2)	0.084 (3)	0.0305 (16)	−0.0347 (19)	−0.0072 (14)	−0.0020 (15)
O5	0.0349 (16)	0.0358 (15)	0.0365 (15)	−0.0001 (12)	0.0004 (11)	−0.0111 (11)
O6	0.0304 (14)	0.0234 (13)	0.0297 (13)	−0.0051 (10)	0.0046 (10)	−0.0019 (10)
O7	0.0483 (16)	0.0218 (12)	0.0159 (12)	−0.0054 (11)	0.0047 (10)	−0.0012 (9)
O8	0.0405 (15)	0.0239 (12)	0.0173 (12)	−0.0002 (11)	0.0096 (10)	0.0007 (9)
O9	0.0332 (15)	0.0246 (13)	0.0314 (13)	−0.0015 (11)	0.0067 (11)	−0.0018 (10)
O10	0.0318 (15)	0.0261 (13)	0.0221 (12)	−0.0036 (11)	0.0029 (10)	−0.0047 (9)
O11	0.0561 (18)	0.0160 (12)	0.0325 (14)	0.0026 (11)	0.0183 (12)	0.0011 (10)
O12	0.0460 (16)	0.0160 (12)	0.0240 (12)	−0.0007 (11)	0.0152 (11)	−0.0013 (9)
O13	0.0280 (16)	0.072 (2)	0.0382 (15)	0.0102 (14)	0.0003 (11)	−0.0260 (14)
O14	0.0296 (18)	0.093 (3)	0.097 (3)	0.0079 (17)	0.0056 (17)	0.062 (2)
C1	0.035 (2)	0.0217 (18)	0.0212 (17)	−0.0014 (15)	0.0080 (14)	0.0011 (13)
C2	0.033 (2)	0.052 (3)	0.0205 (18)	−0.0039 (19)	0.0083 (15)	0.0007 (16)
C3	0.045 (3)	0.052 (3)	0.069 (3)	0.007 (2)	−0.009 (2)	−0.011 (2)
C4	0.029 (2)	0.0226 (18)	0.0282 (18)	−0.0024 (15)	0.0064 (15)	0.0049 (14)
C5	0.030 (3)	0.044 (3)	0.099 (4)	0.002 (2)	0.011 (2)	−0.006 (3)
C6	0.0277 (19)	0.0150 (16)	0.0190 (17)	0.0046 (13)	0.0055 (13)	−0.0003 (12)
C7	0.049 (3)	0.035 (2)	0.033 (2)	−0.0146 (19)	0.0088 (17)	−0.0027 (16)
C8	0.031 (2)	0.029 (2)	0.0175 (16)	0.0030 (16)	0.0068 (13)	−0.0052 (13)
C9	0.040 (3)	0.045 (3)	0.048 (2)	0.010 (2)	0.0007 (19)	0.0001 (19)
C10	0.034 (2)	0.0177 (17)	0.0168 (16)	0.0020 (14)	0.0047 (13)	0.0012 (12)
C11	0.044 (2)	0.032 (2)	0.040 (2)	0.0031 (18)	0.0233 (18)	0.0015 (16)

### Geometric parameters (Å, °)

**Table:**

La1—O3	2.476 (3)	O8—La2^v^	2.739 (2)
La1—O1	2.505 (2)	O9—C8	1.273 (4)
La1—O11^i^	2.515 (2)	O10—C8	1.256 (4)
La1—O2^ii^	2.519 (2)	O11—C10	1.260 (4)
La1—O10	2.548 (2)	O11—La1^iv^	2.515 (2)
La1—O5	2.565 (3)	O12—C10	1.261 (4)
La1—O12	2.572 (2)	O13—H13B	0.8200
La1—O7^iii^	2.578 (2)	O13—H13A	0.8200
La1—O6	2.727 (2)	O14—H14B	0.8733
La2—O4^iv^	2.427 (3)	O14—H14A	0.8611
La2—O6	2.469 (2)	C1—C1^ii^	1.542 (7)
La2—O13	2.517 (3)	C2—C3	1.490 (6)
La2—O8	2.561 (2)	C3—H3A	0.9600
La2—O9	2.598 (3)	C3—H3B	0.9600
La2—O7^v^	2.636 (2)	C3—H3C	0.9600
La2—O12	2.655 (2)	C4—C5	1.484 (5)
La2—O8^v^	2.739 (2)	C5—H5A	0.9600
La2—O11	2.771 (2)	C5—H5B	0.9600
La2—O10	2.802 (2)	C5—H5C	0.9600
O1—C1	1.254 (4)	C6—C7	1.491 (5)
O2—C1	1.247 (4)	C7—H7A	0.9600
O2—La1^ii^	2.519 (2)	C7—H7B	0.9600
O3—C2	1.258 (5)	C7—H7C	0.9600
O4—C2	1.244 (5)	C8—C9	1.498 (5)
O4—La2^i^	2.427 (3)	C9—H9A	0.9600
O5—C4	1.250 (4)	C9—H9B	0.9600
O6—C4	1.272 (4)	C9—H9C	0.9600
O7—C6	1.263 (4)	C10—C11	1.491 (5)
O7—La1^vi^	2.578 (2)	C11—H11A	0.9600
O7—La2^v^	2.636 (2)	C11—H11B	0.9600
O8—C6	1.255 (4)	C11—H11C	0.9600
			
O3—La1—O1	133.67 (9)	C4—O5—La1	99.7 (2)
O3—La1—O11^i^	95.29 (10)	C4—O6—La2	142.5 (2)
O1—La1—O11^i^	83.53 (8)	C4—O6—La1	91.4 (2)
O3—La1—O2^ii^	70.52 (9)	La2—O6—La1	105.00 (8)
O1—La1—O2^ii^	63.98 (8)	C6—O7—La1^vi^	152.2 (2)
O11^i^—La1—O2^ii^	77.69 (9)	C6—O7—La2^v^	98.07 (18)
O3—La1—O10	81.21 (10)	La1^vi^—O7—La2^v^	109.26 (8)
O1—La1—O10	83.72 (8)	C6—O8—La2	146.7 (2)
O11^i^—La1—O10	158.48 (8)	C6—O8—La2^v^	93.35 (18)
O2^ii^—La1—O10	81.16 (9)	La2—O8—La2^v^	118.52 (8)
O3—La1—O5	151.38 (9)	C8—O9—La2	100.4 (2)
O1—La1—O5	73.70 (9)	C8—O10—La1	134.3 (2)
O11^i^—La1—O5	77.68 (8)	C8—O10—La2	91.1 (2)
O2^ii^—La1—O5	132.77 (9)	La1—O10—La2	100.79 (8)
O10—La1—O5	114.99 (8)	C10—O11—La1^iv^	149.3 (2)
O3—La1—O12	78.93 (9)	C10—O11—La2	93.83 (19)
O1—La1—O12	131.63 (8)	La1^iv^—O11—La2	106.98 (8)
O11^i^—La1—O12	135.50 (7)	C10—O12—La1	153.9 (2)
O2^ii^—La1—O12	137.28 (9)	C10—O12—La2	99.38 (18)
O10—La1—O12	64.94 (7)	La1—O12—La2	104.19 (8)
O5—La1—O12	86.80 (8)	La2—O13—H13B	109.5
O3—La1—O7^iii^	78.23 (9)	La2—O13—H13A	139.9
O1—La1—O7^iii^	137.68 (9)	H13B—O13—H13A	110.2
O11^i^—La1—O7^iii^	63.59 (7)	H14B—O14—H14A	123.3
O2^ii^—La1—O7^iii^	127.01 (8)	O2—C1—O1	126.8 (3)
O10—La1—O7^iii^	135.05 (8)	O2—C1—C1^ii^	116.8 (4)
O5—La1—O7^iii^	73.79 (9)	O1—C1—C1^ii^	116.4 (4)
O12—La1—O7^iii^	72.09 (7)	O4—C2—O3	124.0 (4)
O3—La1—O6	138.55 (9)	O4—C2—C3	117.1 (4)
O1—La1—O6	69.50 (7)	O3—C2—C3	118.9 (4)
O11^i^—La1—O6	124.24 (8)	C2—C3—H3A	109.5
O2^ii^—La1—O6	125.26 (8)	C2—C3—H3B	109.5
O10—La1—O6	66.35 (7)	H3A—C3—H3B	109.5
O5—La1—O6	48.66 (7)	C2—C3—H3C	109.5
O12—La1—O6	64.57 (7)	H3A—C3—H3C	109.5
O7^iii^—La1—O6	106.60 (7)	H3B—C3—H3C	109.5
O4^iv^—La2—O6	78.42 (11)	O5—C4—O6	120.1 (3)
O4^iv^—La2—O13	72.15 (10)	O5—C4—C5	119.9 (3)
O6—La2—O13	84.84 (9)	O6—C4—C5	120.0 (3)
O4^iv^—La2—O8	140.15 (10)	O5—C4—La1	56.36 (18)
O6—La2—O8	88.45 (8)	O6—C4—La1	63.83 (18)
O13—La2—O8	69.29 (9)	C5—C4—La1	175.4 (3)
O4^iv^—La2—O9	143.46 (10)	C4—C5—H5A	109.5
O6—La2—O9	113.47 (8)	C4—C5—H5B	109.5
O13—La2—O9	140.44 (8)	H5A—C5—H5B	109.5
O8—La2—O9	76.21 (8)	C4—C5—H5C	109.5
O4^iv^—La2—O7^v^	82.21 (11)	H5A—C5—H5C	109.5
O6—La2—O7^v^	160.30 (7)	H5B—C5—H5C	109.5
O13—La2—O7^v^	92.65 (9)	O8—C6—O7	118.7 (3)
O8—La2—O7^v^	108.97 (7)	O8—C6—C7	120.8 (3)
O9—La2—O7^v^	80.56 (8)	O7—C6—C7	120.5 (3)
O4^iv^—La2—O12	80.35 (9)	O8—C6—La2^v^	62.63 (16)
O6—La2—O12	67.02 (7)	O7—C6—La2^v^	57.95 (16)
O13—La2—O12	144.06 (8)	C7—C6—La2^v^	163.9 (2)
O8—La2—O12	128.57 (7)	C6—C7—H7A	109.5
O9—La2—O12	74.00 (8)	C6—C7—H7B	109.5
O7^v^—La2—O12	106.28 (7)	H7A—C7—H7B	109.5
O4^iv^—La2—O8^v^	117.51 (10)	C6—C7—H7C	109.5
O6—La2—O8^v^	148.30 (7)	H7A—C7—H7C	109.5
O13—La2—O8^v^	75.62 (8)	H7B—C7—H7C	109.5
O8—La2—O8^v^	61.48 (8)	O10—C8—O9	120.6 (3)
O9—La2—O8^v^	71.19 (8)	O10—C8—C9	120.1 (3)
O7^v^—La2—O8^v^	47.50 (7)	O9—C8—C9	119.2 (3)
O12—La2—O8^v^	139.14 (7)	O10—C8—La2	64.91 (18)
O4^iv^—La2—O11	70.01 (10)	O9—C8—La2	55.73 (17)
O6—La2—O11	109.68 (7)	C9—C8—La2	174.4 (3)
O13—La2—O11	135.23 (9)	C8—C9—H9A	109.5
O8—La2—O11	148.94 (8)	C8—C9—H9B	109.5
O9—La2—O11	73.48 (8)	H9A—C9—H9B	109.5
O7^v^—La2—O11	59.48 (7)	C8—C9—H9C	109.5
O12—La2—O11	47.18 (7)	H9A—C9—H9C	109.5
O8^v^—La2—O11	101.74 (7)	H9B—C9—H9C	109.5
O4^iv^—La2—O10	134.52 (9)	O11—C10—O12	119.2 (3)
O6—La2—O10	66.16 (7)	O11—C10—C11	120.4 (3)
O13—La2—O10	128.54 (9)	O12—C10—C11	120.4 (3)
O8—La2—O10	68.43 (7)	O11—C10—La2	62.41 (17)
O9—La2—O10	47.82 (7)	O12—C10—La2	57.11 (16)
O7^v^—La2—O10	128.13 (8)	C11—C10—La2	173.2 (2)
O12—La2—O10	60.43 (7)	C10—C11—H11A	109.5
O8^v^—La2—O10	107.45 (7)	C10—C11—H11B	109.5
O11—La2—O10	95.38 (7)	H11A—C11—H11B	109.5
C1—O1—La1	121.6 (2)	C10—C11—H11C	109.5
C1—O2—La1^ii^	121.1 (2)	H11A—C11—H11C	109.5
C2—O3—La1	117.3 (3)	H11B—C11—H11C	109.5
C2—O4—La2^i^	144.2 (3)		

Symmetry codes: (i) −*x*+1, *y*−1/2, −*z*+3/2; (ii) −*x*+1, −*y*+1, −*z*+2; (iii) *x*, −*y*+3/2, *z*−1/2; (iv) −*x*+1, *y*+1/2, −*z*+3/2; (v) −*x*+1, −*y*+2, −*z*+2; (vi) *x*, −*y*+3/2, *z*+1/2.

### Hydrogen-bond geometry (Å, °)

**Table:**

*D*—H···*A*	*D*—H	H···*A*	*D*···*A*	*D*—H···*A*
O13—H13A···O14	0.82	1.84	2.654 (4)	176.
O13—H13B···O9^v^	0.82	2.01	2.831 (3)	176.
O14—H14B···O9^vii^	0.87	2.12	2.921 (4)	152.9
O14—H14A···O5^viii^	0.86	1.90	2.761 (4)	174.3

Symmetry codes: (v) −*x*+1, −*y*+2, −*z*+2; (vii) *x*−1, *y*, *z*; (viii) −*x*, *y*+1/2, −*z*+3/2.
